# The Effects of Sport Activities and Environmentally Sustainable Behaviors on Subjective Well-Being: A Comparison Before and During COVID-19

**DOI:** 10.3389/fspor.2021.659837

**Published:** 2021-05-24

**Authors:** Mario Wendtlandt, Pamela Wicker

**Affiliations:** Department of Sports Science, Bielefeld University, Bielefeld, Germany

**Keywords:** environment, nature, sport participation, sustainability, well-being

## Abstract

This study examined the effects of sport activities and environmentally sustainable behaviors on the subjective well-being of working-age adults (18–64). Specifically, it analyzes the effects of different types of sport activities, including nature-based, natural resource-using, and nature-neutral sport activities and different types of environmentally sustainable behaviors such as recycling, ecological consumption, energy-saving, and mobility on subjective well-being. The study conducts comparisons between the period before the COVID-19 pandemic and during the first lockdown in Germany. Quantitative survey data were collected using a convenience sampling approach (*n* = 412). Sport activities were captured with the number of hours spent on nature-based, natural resource-using, and nature-neutral activities. Environmentally sustainable behaviors were measured across four areas, including recycling, ecological consumption, energy-saving, and mobility. Subjective well-being was measured using the scale of the World Health Organization (WHO-5). Differences between the periods before and during COVID-19 were analyzed using *t*-tests. A set of multivariate regression models were estimated with subjective well-being as the dependent variable and sport activities, environmentally sustainable behaviors, and socio-demographics as independent variables. The results show that nature-based and nature-neutral sport activities significantly decreased during the first COVID-19 lockdown, while environmentally sustainable behaviors increased. The regression analyses reveal that nature-based and nature-neutral sport activities as well as ecological consumption significantly added to individuals' subjective well-being in the pre- and during-COVID-19-period. A decrease in nature-based and nature-neutral sport activities significantly predicted a decrease in individuals' subjective well-being. The findings of this study might help people understand the role and importance of active living for psychosocial outcomes during the COVID-19 pandemic.

## Introduction

In terms of social policy, environmental protection as well as physical and mental health are important issues. For example, the Green Party was elected in the 2019 European elections with 20.5% of all votes and an increase of 9.8% compared to the 2014 election, signaling an increasing interest in sustainability (State Center for Political Education, 2019). Now, if sustainability is to be promoted, the cumulative effect of individual environmentally sustainable behaviors should not be underestimated (Dietz et al., 2009; EEA, 2015). For example, policy can prescribe guidelines or establish educational programs (Osbaldiston and Schott, 2012). However, if political regulations now determine people's lives, the question arises to what extent these affect the framework for action and an individual's own subjective well-being (SWB).

Thus, it can be assumed that any ordinances that cost people money, time, or effort reduce individual's SWB (Schmitt et al., 2018). However, Schmitt et al. (2018) also provide evidence that ordinances regarding environmentally sustainable behaviors can lead to lifestyle changes that increase rather than decrease individual's SWB. Hence, it can be understood that people are responsible for their own SWB with their individual behaviors. Incentives such as sports and educational programs, clubs and other opportunities can exist, but everyone must initiate their own participation.

Since the beginning of the COVID-19 pandemic, people's lives have changed drastically. To contain the virus, open spaces, restaurants, and sport facilities were closed. People were asked to stay at home and practice social distancing, companies had to reduce working-time or dismiss staff. Questions arise considering the impact of these lifestyle changes on psychosocial factors such as SWB.

The purpose of this study is to examine the effects of sport activities and environmentally sustainable behaviors on individuals' SWB. Specifically, it investigates the effects of different types of sport activities, including nature-based, natural resource-using, and nature-neutral sport activities and different types of environmentally sustainable behaviors such as recycling, ecological consumption, energy-saving, and mobility on the SWB of working-age adults in Germany. It advances two main research questions: (1) how do different types of sport activities and environmentally sustainable behaviors affect SWB? And (2) how do changes in sport activities and environmentally sustainable behaviors between the pre- and during-COVID-19-period impact overall SWB and changes in SWB? These research questions will be analyzed using survey data from Germany.

## Theoretical Framework and Literature Review

### Subjective Well-Being

There are mainly two ways to approach well-being. One the one hand, psychological well-being is a multidimensional construct measuring life aspects such as self-acceptance, positive relations with others, autonomy, environmental mastery, purpose in life, and personal growth (Ryff, 1989; Hernandez et al., 2018). On the other hand, SWB is defined as the cognitive and affective evaluation of individual's own life (Diener et al., 2018). The latter approach to well-being has been chosen to identify the relationship between specific sport activities and environmentally sustainable behavior and a general self-assessed well-being. SWB also includes eudaimonic and hedonic elements. The eudaimonic element means that people strive for self-fulfillment, which motivates them to naturally behave in a way that fulfills their needs. However, Ryan and Deci (2001) point out that some actions are more influenced by norms and external demands, which would not contribute to individuals' SWB. The hedonic element defines SWB as a condition whereby people rather like their life and tend to approach joy and prevent pain (Kahneman et al., 1999).

According to set point theory, individuals' general life satisfaction is genetically anchored (Lykken and Tellegen, 1996) and will be restored eventually to its former level (Brickman and Campbell, 1971). Speaking from a behavioral economics perspective, people naturally behave in a way that would ensure joy and satisfaction which would ultimately contribute to their SWB (Kahneman et al., 1997). Thus, people are acting in a utility oriented and utility maximizing manner. The literature shows that current SWB is driven by individuals' subjective perception and depends on many factors. For example, SWB is related to specific employment factors such as income (Stevenson and Wolfers, 2008; Zhang and Churchill, 2020) or unemployment (Helliwell and Huang, 2014). In addition, associations of SWB with various psychological factors were found, such as depression (Fergusson et al., 2015), personality traits (DeNeve and Cooper, 1998; Steel et al., 2008), goals (Klug and Maier, 2015), prosocial behavior (Thoits and Hewitt, 2001; Dunn et al., 2008), and job satisfaction (Bowling et al., 2010). Specific life events such as weddings, breakups (Luhmann et al., 2012), or parenting characteristics (Nelson et al., 2014) also contribute to changes in SWB. Many different factors and areas of life influence SWB. However, it is not clear which factors stabilize individuals' SWB during life-changing times like the COVID-19 pandemic.

### Sport Activities

Physical inactivity is one of the largest risk factors for global mortality (WHO, 2019). Although there are studies that cannot confirm the relationship between physical activity and health factors (Janssen and LeBlanc, 2010), the majority of studies have indeed shown a positive relationship (e.g., Humphreys et al., 2014; Rhodes et al., 2017; Warburton and Bredin, 2017). For example, cardiovascular diseases, diabetes, cancer, elevated blood pressure, elevated blood glucose levels, and obesity are all associated with physical inactivity. Therefore, strategies and implementation options were developed as early as 2004 in the 57th World Health Assembly Decision and 2008 in the 61st World Health Assembly Decision to provide guidelines and recommendations for physical activity for member countries (WHO, 2010). The German government also recommends a physically active life style for better health, SWB, and quality of life (German Parliament, 2010). For example, the national program “IN FORM - Germany's initiative for healthy nutrition and more exercise” was created (German Parliament, 2010), whereby, among other things, sport activities are promoted in everyday life.

Thus, sport activity is considered a national and global goal for a healthy population, and it is useful to further investigate the relationship of sport with other aspects of life. Rasciute and Downward (2010) showed that people who are more frequently active in sports not only feel healthier, but also happier. Furthermore, sport activities were found to be a positive contributor to SWB (e.g., Pawlowski et al., 2011; Wicker and Frick, 2017; WHO, 2018).

Existing studies have measured (regular) sport activity with a dummy variable (yes or no) (Pawlowski et al., 2011; Huang and Humphreys, 2012; Ruseski et al., 2014) or captured the frequency (Lechner, 2009; Orlowski and Wicker, 2018; Wicker, 2020) or minutes of participation (e.g., Downward and Rasciute, 2011; Wicker and Frick, 2015; Downward and Dawson, 2016). Collectively, these studies found that active people have higher SWB than inactive people and that SWB increases with increasing frequency and minutes of sport activity. Further studies focused on the intensity of sport activities, with higher-intensity activities not significantly contributing to or even reducing SWB (Wicker and Frick, 2015, 2017). Only a few studies examined different types of sport activities. For example, Rasciute and Downward (2010) distinguished between sport activities for health, utilitarian, and competitive purposes.

Existing research has indicated that the context of sport activities matters and the extent to which these are related to nature or consume nature. Regarding nature sports, Bratman et al. (2015) contemplate on general proximity to nature being related to lower distress and higher positive affect. Hence, it is possible that performing sport activities while being connected to nature multiplies those positive effects. Indeed, Wolsko et al. (2019) found that nature-based sport contributes toward more sustainable behaviors and SWB. Nature-based sport includes activities such as walking, cycling, swimming, or snowboarding which do not consume, use, or harm nature in any form. Bratman et al. (2015) also showed that walking in nature reduces rumination, ultimately leading to higher satisfaction and less distress.

On the other hand resource-using sport in nature does not add to SWB (Wolsko et al., 2019). Those activities encompass hunting, fishing, or driving a motorboat which emit carbon dioxide or consume natural resources. It can be concluded that not primarily the connectedness to nature, but rather the cognitive and moral evaluation of the consequences of the respective sport activity is relevant for the influence on SWB. The first hypothesis reflects this relationship:

H1. *The more often individuals engage in nature-based activities, the higher their SWB*.

### Environmentally Sustainable Behaviors

Environmental protection is becoming increasingly important in international politics and for many people. The European Union (EU) has created a package of measures in the context of the European Green Deal. According to this package, Europe attempts to reduce carbon dioxide emissions even further by 2030 and become a climate-neutral continent by 2050 (EU, 2020). Additionally, the European Climate Pact will also be initialized from the last quarter of 2020 to involve all citizens and areas of life in active climate protection. The EU has recognized that measures for the conservation of nature must be defined not only at a political level, but also at an individual level in people's daily lives (e.g., regarding mobility, energy-saving or ecological consumption). Consequently, individual environmentally sustainable behaviors are important. Moreover, studying interrelationships of these behaviors with other aspects of life in more detail is critical as individuals are more likely to perform behaviors which improve their situation and add to their SWB, respectively. Additionally, O'Brien (2008) has pointed out the importance of further investigating the intersection between environmental sustainability and happiness because both aspects can actually benefit from each other in the future.

From a theoretical perspective, a number of theoretical mechanisms explain the association between environmentally sustainable behaviors and SWB (e.g., Schmitt et al., 2018). On the one hand, many types of environmentally sustainable behavior are associated with costs for the individual in terms of, for example, time, money, effort, reduced convenience, or personal sacrifice. The level of these potential costs differs among individuals as every individual has different subjective perceptions in this regard. Depending on the level of these perceived costs, it can be assumed that these costs represent barriers to perform environmentally sustainable behaviors and they explain why performing such behaviors does not improve SWB (Schmitt et al., 2018).

On the other hand, environmentally sustainable behavior is also considered pro-social behavior (Schmitt et al., 2018), meaning that individuals' perceived costs in terms of e.g., time, money, and reduced convenience are accepted to do something good for, in this case, the environment. Accordingly, existing studies have shown that people who behave pro-socially experience positive emotions (Pruneau et al., 2006; Schmitt et al., 2018). This relationship between pro-social behavior and positive emotions can be explained by different psychological mechanisms. For example, people feel proud of themselves when they can help someone or something else through personal sacrifice or at their own expense. This feeling can also be referred to as the warm glow of giving (Andreoni, 1990). Likewise, people often behave altruistically because they want to contribute to the satisfaction of others (Batson and Weeks, 1996; Oliner, 2003). Or they behave less selfishly because they want to reduce their own experience of stress by reducing the suffering of others (Cialdini et al., 1987). Altogether, these mechanisms explain why performing environmentally sustainable behavior can improve individuals' SWB.

From an empirical perspective, many studies have examined the link between environmentally sustainable behaviors and SWB. For example, Wolsko et al. (2019) found that 39 sustainable behaviors positively contribute to individuals' SWB. They conclude that such behaviors facilitate climate protection, especially when nature is endangered due to climate change. This positive relationship between environmentally sustainable behaviors and SWB has also been shown in existing research in the United States (Brown and Kasser, 2005), Mexico (Corral-Verdugo et al., 2011), Sweden (Kaida and Kaida, 2016), Spain (Suárez-Varela et al., 2016), and China (Xiao and Li, 2011). In Germany, Welsch and Kühling (2011) found that recycling, water conservation, and ecological consumption, such as the purchase of environmentally friendly products, were particularly related to individual's SWB. Collectively, these empirical findings support the notion of a positive relationship between environmentally sustainable behavior and SWB, suggesting that the above noted mechanisms of pride, altruism, warm glow, and generosity might be at work. These aspects lead to the second hypothesis:

H2. *The more frequently people perform environmentally sustainable behaviors, the higher their SWB*.

### Behaviors and Subjective

#### Well-Being Before and During COVID-19

Existing research has shown that both sport activities (e.g., Huang and Humphreys, 2012; Ruseski et al., 2014; Downward and Dawson, 2016; Wicker, 2020) and environmentally sustainable behaviors (Schmitt et al., 2018; Wolsko et al., 2019) are positively related to SWB. However, as a result of the COVID-19 pandemic and associated restrictions, these behaviors might have changed and these changes might affect individuals' level of SWB. For example, Evans et al. (2020) argue that the setting of sport activities will change due to the pandemic. So far, Cunningham (2021) found that higher levels of sport activities moderate the relationship between COVID-19 cases and deaths, supporting the importance of analyzing COVID-19 related changes in sport activities and their impact on mental health and SWB, respectively.

The COVID-19 pandemic has changed the private and public lives of everyone around the world. The sport sector was also affected in many ways. For example, sports competitions paused and both fitness clubs and community sport clubs had to close. Government health measures such as social isolation were implemented with the aim of encouraging (or reinforcing) people to stay at home more often. In this time of social isolation and reduced organized sport opportunities, it is possible that people increasingly use walks, runs, bike rides, or (online) home workouts and to what extent these changes in sport activities influence their SWB. For example, Brand et al. (2020) showed that individuals who exercised every day during the pandemic had the best mood, while individuals who decreased their sport activities during the pandemic had the worst mood. Other studies also found positive effects of sport activities on SWB during the pandemic (Lesser and Nienhuis, 2020; Ranasinghe et al., 2020). Again, the cognitive and moral evaluation of sport activities is also believed to influence individuals' SWB during the lockdown (Wolsko et al., 2019). Thus, the third hypothesis reads as follows:

H3. *The greater the increase in nature-based sport, the higher the level of SWB*.

Likewise, lifestyle changes are possible because of an increased number of days working from home or short-time work, allowing people to develop new environmentally sustainable habits (Ramkissoon, 2020). Hence, in light of an increasing awareness of climate protection, the pandemic might have fostered environmentally sustainable behaviors, and an increase in these behaviors might contribute to individuals' SWB. This relationship leads to the fourth hypothesis of this study:

H4. *The greater the increase in environmentally sustainable behaviors, the higher the level of SWB*.

## Methods

### Data Collection

To investigate the research questions, quantitative survey data were collected using a convenience sampling approach in Germany. The online questionnaire was programmed with the internet platform SoSci Survey. The data collection period was from June 1st to August 31st, 2020. The link to the survey was shared with interested participants via private social networks (Facebook, Instagram), professional networks (Xing, LinkedIn) and via e-mail. Additional participants were also recruited via the SurveyCircle and PollPool research platforms. On average, respondents needed about 7 min for the completion of the online questionnaire. The total sample consists of 476 respondents.

Of these 476 respondents, 59 individuals dropped out of the questionnaire early or were non-serious responses (e.g., always medium expression), so these observations were excluded from the dataset. An additional five subjects were removed from the data set because they did not meet the age-based target population. According to the WHO (2010) guidelines, the following age groups are distinctive for considering sport activities and health: 5–17, 18–64, and 65 years and older. The questionnaire was sent to adults between 18 and 64 years old to obtain a sufficiently large sample for the working-age population. Consequently, a total of 412 observations could be included in the empirical analysis.

### Questionnaire and Variables

At the beginning of the survey, respondents were informed about the purpose of the survey, the anonymity of the data collection, and the voluntary nature of participation. The survey consisted of several sections, including sport activities, environmentally sustainable behaviors, SWB, and socio-demographics. All variables used in this study are summarized in Table 1.

**Table 1:** Overview of variables.

**Variable**	**Description**
**SWB (WHO-5; 0** **=** **at no time; 5** **=** **all the time)**	
Good mood	… I was happy and in a good mood
Relaxation	… I felt calm and relaxed
Energy	… I felt energetic and active
Recovery	… I felt fresh and rested when I woke up
Curiosity	… my daily life was full of things that interest me
**Environmentally sustainable behaviors (1** **=** **never; 5** **=** **always)**	
Paper recycling	I recycle paper/newspaper
Glass recycling	I recycle glass
Plastic recycling	I recycle plastic
Organic recycling	I recycle organic material
Packaging in the store	I leave the packaging material in the shopping stores where I bought the products
Eco label	I buy products with an eco-label
Seasonal food	I buy seasonal and regional fruits and vegetables
Refillable bottles	I buy beverages in refillable or recyclable bottles
Turn off light	I turn off the lights when I walk out of a room in my home
Energy-saving bulb	I use energy-saving lamps in my household
Water-saving devices	I use appliances in my household that use less water (e.g., water-saving shower head)
Turn off water	When I shower or bathe, I turn off the water when I soap myself with shower gel/shampoo
Shopping	I go shopping without a car
Weekend trips	I plan my weekend trip so that I don't need a car
Traveling	I reach my destinations on vacation without a car, plane or cruise ship
Car usage in the household	I use a car in my household (reverse coded)
**Sport activities (hours per week)**	
Nature dependent sports	Canoeing, kayaking, skiing, snowboarding, stand-up paddling, swimming in an outdoor pool or the ocean
Nature independent sports	Hiking, horseback riding, golf, running, biking, ball sports
Consuming sports	Hunting or fishing
Motorized sports	Quad bike, motor boat
Nature-neutral sports	Gymnastics, basketball, handball, tennis, soccer, fitness training at home or in a gym, swimming in an indoor pool
**Employment**	
Working time	Number of weekly working hours
Working time^2^	Working time*Working time
Home office	Number of weekly days in home office
Income	Personal net income per month (from 1 = €500 to 9 = >€4000)
**Sociodemographic variables**	
Woman	Respondent's gender (0 = man, 1 = woman)
Age	Respondent's age (in years)
Age^2^	Age*Age
Low educational attainment	0 = no, 1 = yes
Higher education entry qualification	0 = no, 1 = yes
University degree	0 = no, 1 = yes
State	Dummies for the 16 German states

Within the section assessing sport activities, questions related to the weekly number of hours spent with different forms of sport activities were asked for both the pre-COVID-19-period (i.e., 2019) and the during-COVID-19-period (i.e., 2020). The different types of sport activities were measured using an existing typology. According to Wolsko et al. (2019), sport activities can be classified into nature-dependent outdoor activities (e.g., canoeing, kayaking, skiing, snowboarding, stand-up paddling, swimming in an outdoor pool or the ocean), nature-independent outdoor activities (e.g., hiking, horseback riding, golf, running, cycling, or ball sports), consumptive nature-related sports (e.g., hunting or fishing), and motorized activities in nature (e.g., quad bikes, motorboats). The category nature-neutral sports (e.g., gymnastics, basketball, handball, tennis, soccer, fitness training at home or in a gym, and swimming in an indoor pool) was added as well as the option to indicate other activities. Nature-dependent and nature-independent outdoor activities neither pollute nor consume nature, so these two categories are combined as nature-based activities. Consuming nature-related sports and motorized activities in nature, on the other hand, pollute nature or cause noise and are thus summarized as resource-using activities. If respondents listed further activities in the category “other activities,” they were manually assigned to the appropriate main categories (if possible) or were excluded when they were not considered sport (e.g., gardening). No further sport activity category emerged from the category “other.” Overall, the sport activities variables reflect the number of weekly hours respondents spend on the respective sport activities.

Environmentally sustainable behaviors pre (2019) and during the COVID-19 pandemic (2020) were assessed with a set of items. Specifically, environmentally sustainable behaviors were classified into four categories, including recycling, ecological consumption, energy-saving, and mobility (Diekmann and Preisendörfer, 2003). For recycling, a distinction was made between paper/newspaper, glass, plastic, and organic/waste residues. Each category was captured by four items which were provided to respondents in randomized order. All items were measured on a five-point Likert scale. For reasons of comparability, the four-point Likert scale of Diekmann and Preisendörfer (2003) was not used here, but rather a five-point scale since the other constructs were also assessed using a five-point Likert scale.

The four latent factors recycling, ecological consumption, energy-saving, and mobility are not directly measurable, but were used as recommended by Diekmann and Preisendörfer (2003). To assess the dimensionality of the scale, a confirmatory factor analysis was run. The loadings vary between 0.28 and 0.88, with most of the variables being in the acceptable range. Even if a few single loadings are weak, the variables are not removed from the model because they still indicate the same direction of effect (Hair et al., 2010). With the comparative fit indexes (CFIs) > 0.89, the standardized root-mean square residuals (SRMR) < 0.10, and the root mean square errors of approximation (RMSEA) < 0.10, all criteria for a good model fit are met. Thus, it can be concluded that the variables meaningfully represent the four latent factors (Kearney, 2006). The factor analysis was only conducted for assessing the dimensionality of the scale. Both the *t*-test and the regression analysis are based on the four mean variables capturing the four types of environmentally sustainable behavior. The reliability of the scale was assessed using Cronbach's alpha. The corresponding values for the 2019 and 2020 data are α = 0.75 and α = 0.77, respectively. According to Taber (2018), values above 0.70 are considered satisfactory.

Respondents' SWB pre (2019) and during the COVID-19 pandemic (2020) was assessed using the WHO-5 scale which was developed by the World Health Organization (1998). This scale has already been translated into more than 30 languages and has been validated many times (e.g., Topp et al., 2015; Zeike et al., 2019). The scale contains of five items. The items are positively worded and relate to mood, vitality, and general interest in individual's own life over the past 2 weeks. They are measured on a 6-point scale. A confirmatory factor analysis was calculated to evaluate the reliability and dimensionality of the five variables being summarized as a latent factor of SWB. Due to consistently high loadings of the variables (>0.70) on the SWB factor and very good fit indices (*CFI* > 0.95) for both models, unidimensionality can be assumed. The Cronbach's alphas are α = 0.87 and α = 0.91 for the 2019 and 2020 data, respectively, indicating strong internal consistency (Taber, 2018). The five items of the WHO-5 scale are added up to a sum score, which is further multiplied by four, analogous to the study of Topp et al. (2015). Hence, the SWB variable has a range from 0 to 100.

Dolan et al. (2008) summarized the effects of employment-related and sociodemographic factors on SWB. These factors are included as control variables in the present analysis. Employment in the form of average working time, the number of days working from home and income at the time before the pandemic in 2019 and after the first lockdown in 2020 was assessed to see whether a change in employment conditions influenced an individual's SWB. Working time was measured by the average number of working hours per week and is expected to have a u-shaped relationship with SWB. To capture these possible non-linear effects, squared working time was included in the analysis (Dolan et al., 2008). Home office was measured by the average number of days worked from home and is expected to be moderately related to SWB (Hayman, 2010). Concerning income, respondents could choose between nine categories with intervals of €500. Several studies so far showed that higher income also contributes to higher SWB (for a review see Dolan et al., 2008).

According to Babbie (2010), the inclusion of sociodemographic factors such as age, gender, and educational level as control variables in regression analyses enhances the generalizability and interpretability of findings. Previous research on the relationship between age and SWB found a u-shaped relationship (e.g., Dolan et al., 2008; Ruseski et al., 2014). Gender was coded as a dummy variable, with men being assigned a zero and women assigned a one. Two individuals assigned themselves to neither male nor female gender. These were not considered further due to the small number of cases. While some studies found that women reported higher SWB than men (DiTella et al., 2003; Huang and Humphreys, 2012), others did not find any gender differences in SWB (Louis and Zhao, 2002; Schmitt et al., 2018). Regarding educational level, it can be expected that educational attainment indirectly influences SWB by being related to a higher standard of living, more income, and health (DiTella et al., 2003; Dolan et al., 2008). Therefore, the educational level is also included in the regression as a dummy variable. Like in the study of Wicker (2020), educational attainment is recoded into three dummy variables reflecting low educational attainment (some form of secondary school; i.e., below A-levels), higher education entry qualification (i.e., technical college or university entry qualification), and university (i.e., university degree).

Additionally, the state where respondents live is also assessed in the survey and included in the regression as a dummy variable to investigate whether respondents' place of residence has a significant influence on SWB. One study has detected differences in SWB between East and West Germany (Schimmack et al., 2008). The variable federal state was recoded from 16 to eleven categories, since fewer than ten respondents came from the federal states of Brandenburg, Bremen, Mecklenburg-Western Pomerania, Saarland, Saxony, and Saxony-Anhalt. These were collapsed into the category “other.” The reference category is North Rhine-Westphalia.

### Data Analysis

This study aimed to analyse, first, whether the sport activities, environmentally sustainable behaviors and SWB changed between 2019 and 2020 using *t*-tests and, second, the effect of sport activities and environmentally sustainable behaviors on SWB using regression analyses. Paired samples *t*-tests were employed to analyse differences in sport activities, environmentally sustainable behaviors, SWB, and employment conditions between the pre-COVID-19-period and the during-COVID-19-period. Altogether, four linear regression models were estimated. The first model is for the pre-COVID-19-period (2019 data), while the second model includes the during-COVID-19-period (2020 data). The third model examines how changes in sport activities and environmentally sustainable behaviors affect changes in SWB, implying that difference variables are included in this model. The last model also includes changes in sport activities and environmentally sustainable behaviors, but the dependent variable is SWB during the pandemic (i.e., in 2020).

For the regression analysis to be employed, certain prerequisites and conditions were met. The first condition was to thoroughly check the data set for outliers and influential data points to be able to exclude measurement errors of the predictors and the dependent variable (Eid et al., 2017). Overall, all measurement values that were impossible to obtain were removed. All other values that were within the realistic range were retained. The second requirement for multiple regression analysis was the exclusion of multicollinearity of predictors, which means that the independent variables must meaningfully and independently contribute to variance explanation in SWB and should not be highly correlated with each other, otherwise, the regression weights can only be estimated imprecisely (Eid et al., 2017). Multicollinearity was tested using correlation analysis. All correlation coefficients were below 0.8 and all variance inflation factors were <10 (except for age and squared age and work time and squared work time), suggesting that multicollinearity of predictors was not an issue in the present analysis (Hair et al., 2010).

The second pre-condition was normal distribution of residuals which was required to accurately estimate the standard errors (Eid et al., 2017). This assumption was tested using Q-Q-plots which showed that the assumption was not violated. The third pre-condition was the homoscedasticity of the residuals which was analyzed with scale-location plots. These plots gave relatively uniform standard deviations of the residuals over the range of the dependent variable (normalized between 0 and 100), meaning this condition was also not violated. Thus, the regression model made equally good predictions for all values and respondents. Another pre-condition was linearity of relationships, which was tested and confirmed using scatterplots. Finally, for an unbiased multiple regression analysis, the residuals of the variables were also independent of each other (Eid et al., 2017). The Durbin-Watson coefficient was *DW* = 2.01 (*p* = 0.970) for the 2019 model and *DW* = 2.11 (*p* = 0.318) for the 2020 model. Also, the non-significant *p*-value showed that there was no autocorrelation of the residuals. The condition of independent residuals was hereby fulfilled. Hence, all pre-conditions for applying regression analysis were met.

## Results

At the time of the survey, respondents were 27 years old on average. The youngest respondent is 18 years and the oldest is 64 years old. Female respondents make up two thirds of the sample (66.75%). Overall, just under 60% of respondents have a degree from a university or technical college and just under 37% have a technical college or university entry qualification.

Table 2 shows the summary statistics of SWB for 2019 and 2020 as well as the results of the *t*-tests. Respondents' self-reported SWB reveals that, on average, they felt positive slightly more than half of the time in a typical week in 2019 (*M* = 86.76). On average, subjects feel less positive about their situation in 2020 than in 2019 (*M* = 78.47). The average reduction in SWB is highly significant (*p* < 0.001).

**Table 2:** Well-being in 2019 and 2020 (*n* = 412).

	**2019**		**2020**		**Difference 2020–2019 (*****t*****-test)**		
**Item (0 = at no time; 5 = all the time)**	***M***	**SD**	***M***	**SD**	***M***	***t***	***p***
Good mood	18.34	3.61	16.64	4.49			
Relaxation	17.39	4.20	15.62	5.15			
Energy	17.43	4.24	15.58	4.87			
Recovery	16.43	4.84	14.83	5.22			
Curiosity	17.17	4.32	15.80	4.90			
SWB	86.76	17.29	78.47	21.04	−8.282	−8.537	<0.001^***^

*** *p < 0.001*.

Table 3 summarizes the descriptive statistics for environmentally sustainable behaviors and the results of the *t*-tests. They reveal that respondents often behave in an environmentally sustainable manner. The mean value of the recycling factor is *M* = 3.72 in 2019 and increases to *M* = 3.90 in 2020. For ecological consumption, the mean increases from *M* = 3.06 in 2019 to *M* = 3.17 in 2020. For mobility, the mean increases from *M* = 2.90 to *M* = 2.99 during the same period. A slight average increase in environmentally sustainable behaviors is also seen for energy conservation, with a mean of *M* = 3.49 in 2019 and of *M* = 3.55 in 2020. Altogether, in 2020, all environmentally sustainable behaviors were performed more frequently than in 2019. The pairwise tests show that these differences are statistically significant.

**Table 3:** Environmentally sustainable behaviors in 2019 and 2020 (*n* = 412).

	**2019**		**2020**		**Difference 2020–2019 (*****t*****-test)**		
**Item (1 = never; 5 = always)**	***M***	**SD**	***M***	**SD**	***M***	***t***	***p***
Paper recycling	3.83	1.29	4.06	1.16			
Glass recycling	4.03	1.18	4.12	1.19			
Plastic recycling	3.80	1.29	3.93	1.20			
Organic recycling	2.23	1.34	3.50	1.36			
Recycling (mean)	3.72	1.05	3.90	1.05	0.181	7.283	<0.001^***^
Packaging in store	2.07	1.08	2.15	1.15			
Eco-label	3.13	0.99	3.25	0.99			
Seasonal food	3.46	0.93	3.60	0.89			
Refillable bottles	3.60	1.20	3.70	1.24			
Ecological consumption (mean)	3.06	0.70	3.17	0.71	0.110	5.082	<0.001^***^
Switch off lights	4.43	0.81	4.44	0.79			
Energy saving light bulbs	3.81	1.15	3.85	1.19			
Water saving devices	2.45	1.22	2.55	1.28			
Turn off water	3.28	1.54	3.37	1.55			
Energy-saving (mean)	3.49	0.76	3.55	0.79	0.059	3.433	<0.001^***^
Shopping	3.34	1.35	3.31	1.36			
Weekend getaway	2.86	1.22	2.92	1.26			
Travel	2.26	1.23	2.53	1.32			
Car in the household (reverse coded)	3.14	1.38	3.18	1.43			
Mobility (mean)	2.90	0.58	2.99	0.64	0.065	2.054	<0.001^***^

*** *p < 0.001*.

Table 4 shows the summary statistics for sport activities and the results of the *t*-tests. Nature-dependent sport activities outside were performed more frequently in 2019 (*M* = 1.69) than in 2020 (*M* = 1.07). In 2020, the frequency of nature-independent activities outside also decreased by 0.02 h per week compared to 2019. Thus, in 2020, respondents reported spending an average of 4.54 h per week of time hiking or similar activities. On average, nature-based sport was thus practiced more frequently in 2019 before COVID-19 (*M* = 6.25) than during the pandemic in 2020 (*M* = 5.62).

**Table 4:** Sport activities in 2019 and 2020 (*n* = 412).

	**2019**		**2020**		**Difference 2020-2019 (*****t*****-test)**		
**Sport activities (in hours per week)**	***M***	**SD**	***M***	**SD**	***M***	***t***	***p***
Nature dependent sport	1.69	7.53	1.07	2.89			
Nature independent sport	4.56	5.62	4.54	5.16			
Nature-based sport	6.25	9.75	5.62	5.95	−0.619	−1.369	0.172
Consuming sport	0.41	5.98	0.55	7.54			
Motorized sport	0.28	1.65	0.25	1.64			
Natural resource-using sport	0.69	6.24	0.80	8.58	0.112	0.616	0.539
Nature-neutral sport	3.08	3.32	2.77	3.46	−0.312	−2.043	0.042*

*** *p < 0.001*.

In 2020, respondents engaged more frequently in hunting or fishing (*M* = 0.55) than in 2019 (*M* = 0.41). Motorized activities outside decreased by an average of 0.03 h per week. Overall, resource-using sport activities increased by an average of 0.11 h per week compared to 2019. The second most common sport activity was nature-neutral sport. On average, respondents dedicated 0.31 more hours to indoor sport activities per week in 2019 (*M* = 3.08) than in 2020 (*M* = 2.77). Overall, the *t*-tests reveal that only nature-neutral sport activities have significantly changed between 2019 and 2020.

Table 5 displays the descriptive statistics and the results of the *t*-test for the employment variables. During the pandemic in 2020, respondents worked an average of 3 days per week in the home office. Before COVID-19, they were in home office only between 1 and 2 days per week. In total, respondents worked just under 20 h per week both before COVID-19 and in 2020, including work in home office and at the normal workplace. The high standard deviation of the working hour's variable can be explained by some extreme values that were not removed because respondents explained in the text box that these many overtime hours were operationally necessary and thus realistic. The mean personal net income is between €500 and €1,500 per month in both 2019 and 2020.

**Table 5:** Employment situation in 2019 and 2020 (*n* = 412).

	**2019**		**2020**		**Difference 2020–2019 (*****t*****-test)**		
	***M***	**SD**	***M***	**SD**	***M***	***t***	***p***
Working time	19.97	17.73	19.58	18	−0.039	−0.557	0.578
Home office	1.58	2.24	2.96	2.81	1.355	10.520	<0.001^***^
Income	2.78	1.92	2.80	2.04	0.03	0.549	0.584

*** *p < 0.001*.

Table 6 presents the results of the multiple regression analysis. Starting with sport activities, the 2019 model shows that nature-based sport activities are significantly related to SWB, but resource-using sports do not. Nature-neutral sport activities are also significant. Nature-based sport activities are also a significant predictor of SWB for the period during the pandemic in 2020. Thus, SWB increases by slightly more than one unit when people perform more hours of nature-based sport (ceteris paribus [c.p.]). Resource-using sport continues to be non-significantly related to SWB. However, nature-neutral sport is again a significant predictor of SWB (c.p.). It was hypothesized that more frequent engagement in nature-based sport activities (hypothesis H1) would be related to higher SWB. Overall, the results show that nature-based sport activities are significantly and positively related to SWB, suggesting that the first hypothesis can be supported.

**Table 6:** Regression analyses for SWB (*n* = 412).

	**SWB 2019**	**SWB 2020**	**SWB Diff 20-19 (IVs: Diff 20-19)**	**SWB 2020 (IVs: Diff 20-19)**
Constant	57.940^***^	43.790^*^	2.833	69.782^***^
	(16.680)	(20.300)	(18.887)	(20.85)
Recycling	2.095^*^	1.841	−4.317^*^	−1.137
	(0.922)	(1.112)	(2.102)	(2.321)
Ecological consumption	2.734^*^	4.339^*^	0.263	4.738
	(1.384)	(1.806)	(2.477)	(2.735)
Energy-saving	2.257	2.090	5.674	5.018
	(1.219)	(1.486)	(3.180)	(3.512)
Mobility	−1.274	0.495	0.012	0.813
	(0.907)	(1.056)	(1.670)	(1.844)
Nature-based sport	0.218^*^	0.519^**^	0.356^**^	0.197
	(0.098)	(0.180)	(0.129)	(0.143)
Resource-using sport	−0.004	0.066	0.177	0.155
	(0.133)	(0.122)	(0.352)	(0.388)
Nature-neutral sport	0.542	0.903^**^	1.065^**^	0.656
	(0.282)	(0.315)	(0.370)	(0.408)
Age	−0.542	−1.119	−0.413	−0.563
	(0.969)	(1.181)	(1.111)	(1.228)
Age^2^	0.009	0.016	0.008	0.012
	(0.013)	(0.016)	(0.016)	(0.017)
Woman	−0.642	−5.916^*^	−5.631^*^	−4.232
	(1.932)	(2.349)	(2.192)	(2.421)
Working time	−0.038	−0.043	0.280	0.220
	(1.214)	(0.154)	(0.178)	(1.251)
Working time^2^	0.000	−0.001	−0.007^*^	−0.005
	(0.002)	(0.002)	(0.003)	(0.004)
Income	0.836	1.419	−0.836	0.211
	(0.671)	(0.728)	(1.133)	(1.251)
Home office	−0.316	0.120	−0.284	−0.808
	(0.412)	(0.441)	(0.453)	(0.500)
Low educational attainment	−10.550	−1.074	12.026	−0.949
	(6.605)	(7.442)	(7.658)	(8.457)
Higher educational entry qualification	−3.269	−2.277	−0.195	−2.529
	(2.021)	(2.539)	(2.424)	(2.677)
State	Yes	Yes	Yes	Yes
R^2^	0.166	0.176	0.171	0.124
Adj. R^2^	0.102	0.112	0.104	0.052

* *p < 0.05*;

** *p < 0.01*;

*** *p < 0.001*;

*IVs = independent variables; displayed are the unstandardized regression coefficients; standard errors in parentheses; reference categories are male, university degree, and North-Rhine-Westphalia*.

Turning to environmentally sustainable behaviors, the results of the 2019 model show that recycling, ecological consumer behaviors and energy-saving behaviors as well as nature-based sports are significant predictors of SWB. Overall, a one-unit increase in recycling behaviors ceteris paribus (c.p.) leads to a 2.095 unit increase in SWB. SWB also increases by 2.734 units when ecological consumption behaviors increase by one unit (c.p.). Energy-saving behaviors are a significant predictor of SWB, while mobility behaviors are not. Hypothesis H2 can thus be partially confirmed, as individual environmentally sustainable behaviors are positively related to SWB. Some theory-conforming significant correlations can also be identified in the 2020 model. Ecological consumption behaviors show to be a suitable predictor (c.p.) for SWB in this model. Recycling behaviors are only marginally significant, while energy-saving and mobility behaviors are not significantly related to SWB in the 2020 model. It was hypothesized that more environmentally sustainable behaviors (hypothesis H2) would be related to higher SWB. The results show that individual environmentally sustainable behaviors (ecological consumption and recycling) are significantly and positively related to SWB, so the second hypothesis can be partially supported.

The next step was to analyse how behavioral changes affect SWB. The results of the multiple regression analysis with difference terms show that a change in recycling behaviors, nature-based sports, and nature-neutral sports are significantly related to a change in SWB. If the 2020-2019 difference in recycling behaviors increases by one unit, SWB decreases by 4.137 units. Energy conservation is marginally significant. If the difference in nature-based behaviors increases by one unit from 2020 and 2019, the difference in SWB increases by 0.712 units. The change in nature-neutral sports is also a significant predictor of the change in SWB. In this study, SWB during the pandemic in 2020 cannot significantly be predicted by a change in the independent variables.

Regarding behavioral changes, it was hypothesized for the comparison before COVID-19 and during COVID-19 that increases in nature-based sport activities (hypothesis H3) and environmentally sustainable behaviors (hypothesis H4) would also be positively related to SWB. Hypothesis H3 can be supported because the increase in nature-based sport activities is significantly and positively related to an increase in SWB. However, the results of the difference analysis show that SWB decreases significantly despite an increase in recycling behaviors and that an increase in environmentally sustainable behaviors does not yield increases in SWB. Therefore, hypothesis H4 must be rejected.

## Discussion

This study looked at the effects of different types of sport activities and environmentally sustainable behaviors on individuals' SWB. For this purpose, a quantitative survey was designed and data were collected in Germany. Due to the private and professional life changes because of the COVID-19 pandemic and the resulting lockdown, both the situation before COVID-19 (in 2019) and the situation during the pandemic (in 2020) were assessed in the survey. In summary, both in retrospect in 2019 and the evaluation of the current situation, individual environmentally sustainable behaviors and nature-based sports were found to be related to SWB.

### Discussion of Findings

The first hypothesis was that sport activities that are nature-based positively contribute to SWB. The results of the regression analysis reflect that nature-based sport activities correlate more strongly with SWB than resource-using activities (e.g., hunting, fishing, motor boating, or quad biking). In both the 2019 and 2020 models, nature-based sport activities are a positive predictor of SWB, even when controlling for employment and sociodemographic variables. Hence, performing these activities contributes to individuals' SWB because the health and social benefits of sport activity do not occur at the expense of the natural environment. Nature-based sport activities such as cycling, running, swimming, or skiing do not consume any natural resources and do not cause any negative consequences such as noise or pollution. Beyond noise or pollution, the impact of skiing on nature can be seen controversially as skiing pistes and resorts can also damage the natural environment (Digel, 2013). Acknowledging this controversy, it was nevertheless included in the nature-based sport activity category as proposed by Wolsko et al. (2019). Moreover, nature-neutral sport activities were a significant determinant of SWB in 2020 and increasing the duration of these activities from 2019 to 2020 also yielded increases in SWB. Hence, also sport activities that do not consume or harm the natural environment help people to feel better. Only the feeling of consuming or harming environmental resources during sport activities do not impact SWB. The finding that duration of sport activities positively affects SWB is in line with existing research (e.g., Wicker and Frick, 2015; Downward and Dawson, 2016), which, however, did not distinguish between different types of sport activities. The contribution of the present work lies in a nuanced analysis of sport activities and a classification of these activities based on their relation to nature.

The second hypothesis stated that people with more pronounced environmentally sustainable behaviors also experience greater SWB. Schmitt et al. (2018) showed that almost all environmentally sustainable behaviors are related to individual's life satisfaction. The results of this study can partially confirm this relationship. Ecological consumption is a significant predictor of SWB in both models (2019 and 2020). Recycling was also a suitable behavior to improve individuals' SWB in 2019. Mobility and energy-saving behaviors, on the other hand, were not significantly related to SWB.

The third and fourth hypotheses were related to changes in sport activities and environmentally sustainable behaviors and how these changes affect SWB in 2020 or changes in SWB between 2019 and 2020. In particular, due to the COVID-19 pandemic, there were some restrictions, bans, and regulations that changed people's daily life as early as March 2020. The *t*-test supports the notion that the pandemic has changed various parts and activities of people's lives, including environmentally sustainable behaviors, nature-neutral sport activities, SWB, and work in home office. The significant decline in nature-neutral sport activities can be explained by the closure of many sport facilities, including especially indoor facilities, but also outdoor facilities to some extent depending on the respective lockdown regulations. On the contrary, participation in nature-based and natural-resource using sport activities did not change significantly. It is possible that interested individuals did not have the opportunity to participate in these activities during the pandemic as organizations offering these sport activities were also closed and/or travel restrictions were in place.

The question is to what extent sport activities or environmentally sustainable behaviors can serve as resilience factors for alleviating the negative well-being impacts on a crisis such as the COVID-19 pandemic. The COVID-19 pandemic and everyday lifestyle restrictions are shaping people's lives and preferences for future sport activities (Teare and Taks, 2021). For example, because of COVID-19 related restrictions, people were found to spend less time in outdoor recreational settings (Landry et al., 2021). It is possible that the increased accessibility of digital sport offers, and the lack of offline sport opportunities are responsible the positive effect of nature-neutral sport activities on SWB. Qin et al. (2020) have identified less physical activity and increased screen time to be related to poorer emotional state during the nationwide lockdown in China in spring 2019. Cindrich et al. (2021) have also found that during COVID-19 decreasing hours spent outside led to more negative stress and lower positive mental health levels. Both aspects, sport activities themselves and time in nature, were identified as important factors for SWB in the present work as well. While nature-neutral sport activities were not relevant to SWB before the pandemic, they gained in importance during the pandemic because of stay-home policies and were able to add to people's SWB. Nature-based sport activities also significantly added to SWB, while it was still important to not harm nature.

Binder and Blankenberg (2017) found that self-assessed sustainability of an individual's behaviors are strongly related to individual's life satisfaction, precisely because reflection on the positive impact on the environment generates satisfaction. Thus, the cognitive focus people have while assessing their SWB is critical. Loewenstein and Schkade (1999) call this phenomenon focusing illusion. It may be that the focus during the COVID-19 pandemic is not on sustainability, but on health and occupational safety. This may explain why SWB decreases in this data set, although environmentally sustainable behaviors increase. In accordance with set point theory, it can be assumed that individuals' average SWB will improve again, even if the pandemic continues for a longer time. This adjustment tends to happen because people adapt to this adverse situation and shift their cognitive focus to other aspects of their life. Still, being physically active was found to support individuals in bouncing back from adversity more quickly than without participating in sport and physical exercise (Wicker and Orlowski, 2020).

Sociodemographic factors such as age, gender, level of education and state, as well as employment factors such as working time, income, and home office were included to control for other factors that also affect individuals' SWB and, in doing so, isolate the effects of sport activities and environmentally sustainable behaviors. The self-assessment of the situation during the pandemic (2020) revealed that women reported lower SWB levels than men. This gender difference in favor of men echoes exiting research (e.g., Dolan et al., 2008) and suggests that the typical drivers of SWB are also evident in a crisis situation like the COVID-19 pandemic. In the present study, women also experienced significantly larger declines in SWB during the pandemic compared too before the pandemic. This finding differs from Lades et al. (2020) who found no gender differences in daily well-being outcomes during the COVID-19 pandemic. The other socio-demographic factors played no role in either model.

The increased number of days people worked from home indicate that the pandemic has significantly changed their working conditions. This finding echoes Brynjolfsson et al. (2020) who have reported that at least one third of the labor force in the United States have changed to remote work between February and May 2020. Such changes can be perceived as financially or organizationally inconvenient, hindering or endangering, and could reduce people's SWB because they are worried about their professional future or their finances. However, the present study supports the notion that these changes in the working environment did not affect their SWB, meaning that they could also be considered by employers in the period after the pandemic.

### Limitations

The first limitation of the study is the generalizability of the data. Looking at the sample, it is noticeable that almost 85% of respondents are under 30 years old and around two thirds are women. This gender and age composition indicates that the sample is not representative of the German resident population and it might have skewed the results. This high proportion of younger people can be explained by the fact that the personal network and the people using platforms such as Survey Circle or PollPool are younger. The second limitation is that the different types of environmentally sustainable behaviors were not weighted depending on their (perceived) threat to the natural environment. According to Schmitt et al. (2018), this leads to more environmentally sustainable behaviors. It can be assumed that those individuals who perceive the environment to be threatened feel happier when they engage in environmentally sustainable behaviors that greatly reduce environmental impact. However, it is unclear whether the perception of environmental threat has decreased, increased, or remained the same during the COVID-19 pandemic compared to before the pandemic as this aspect was not included in the survey. The third limitation considers the retrospection of behaviors and SWB in 2019 and the potential of recall bias. Some participants might have had difficulties in accurately recalling their sport activities, environmentally sustainable behaviors, and SWB in the time period before the pandemic.

### Future Research Directions

In this study, sport activities were classified based on the connectedness to nature, yielding a distinction between sports related to nature and sports neutral to nature. Schmitt et al. (2018) mention in their study that, in addition to pure nature-relatedness, social connection plays an important role in SWB. Another perspective for future studies would be to differentiate based on social connection within nature-neutral and nature-related sport activities. Social connection in sport activities that are conducted within groups represents an opportunity for prosocial behavior and exchange of knowledge, which can also affect SWB. While organized recreational sports in group settings paused during the lockdown, people in a social space such as a digital training group might engage more with other participants.

The present study relied on quantitative survey data with a retrospective part. It is possible that respondents had difficulty recalling their level of sport activities and environmentally sustainable behaviors. Follow-up studies should examine the relationship between sport activities, environmentally sustainable behaviors, and psychosocial factors such as SWB using longitudinal research designs where individuals' behaviors and SWB levels are recorded over a longer period of time. Naturally, with longitudinal studies, the anonymity of participants is compromised.

This interdisciplinary study focused on the intersection of sports sciences, health/psychology, and environmental sustainability to examine the relationship between individual behaviors and SWB. A positive correlation of sport activities, environmentally sustainable behaviors and SWB is of high relevance for climate change and human health at the same time. In summary, people feel better overall when they include environmentally sustainable behaviors or nature-based outdoor sport activities in their life. With the backdrop of increasing “green politics” (State Center for Political Education, 2019), public interest in sustainability, resource conservation, and environmentally sustainable behaviors is on the rise. Accordingly, future research should continue to approach this topic from interdisciplinary perspectives. The present research design of studying nature-related activities and environmentally sustainable behaviors could also be applied to other contexts of daily life such as work, education, and leisure tourism.

## Data Availability Statement

The raw data supporting the conclusions of this article will be made available by the authors, without undue reservation.

## Ethics Statement

Ethical review and approval was not required for the study on human participants in accordance with the local legislation and institutional requirements. Written informed consent for participation was not required for this study in accordance with the national legislation and the institutional requirements.

## Author Contributions

MW designed the survey, collected the data, and drafted the first version of the paper. PW oversaw the data collection process, revised and partially rewrote paragraphs in all chapters, and checked the overall manuscript for coherence, consistency, and format. Both authors contributed to conception and design of the work, drafted it, and revised it critically for important intellectual content, approved the final version of the manuscript, agree to be accountable for all aspects of the work in ensuring that questions related to the accuracy or integrity of any part of the work are appropriately investigated and resolved. All persons designated as authors qualify for authorship, and all those who qualify for authorship are listed.

## Conflict of Interest

The authors declare that the research was conducted in the absence of any commercial or financial relationships that could be construed as a potential conflict of interest.
